# Guarding the heart in the era of immunotherapies: insights for cardio-oncology practice

**DOI:** 10.3389/fphar.2026.1762239

**Published:** 2026-01-26

**Authors:** Umamah Iram, Djamel Lebeche

**Affiliations:** 1 Department of Physiology, The University of Tennessee Health Science Center, Memphis, TN, United States; 2 Department of Medicine, The University of Tennessee Health Science Center, Memphis, TN, United States; 3 Neuroscience Institute, College of Medicine, The University of Tennessee Health Science Center, Memphis, TN, United States

**Keywords:** cancer immunotherapy, cardio-oncology, cardiotoxicity, chimeric antigen receptor T-cell therapy, immune checkpoint inhibitors

## Abstract

The advent of cancer immunotherapies, including immune checkpoint inhibitors (ICI), Chimeric antigen receptor (CAR-T) cell therapies, and bispecific antibodies and other immune-activating platforms such as therapeutic vaccines and oncolytic viruses (often used in combination regimens) has revolutionized oncology by improving patient outcomes across diverse malignancies. However, these therapies are increasingly linked to cardiovascular toxicities such as myocarditis, arrhythmias, and heart failure, posing significant clinical challenges. Here we review current evidence on the mechanisms, clinical manifestations, diagnostic approaches, and management strategies of immunotherapy-associated cardiotoxicities, emphasizing the role of cardio-oncology in integrating cardiovascular care with cancer treatment. We also discuss emerging immunotherapies and their potential cardiac effects. Understanding these complexities is critical to optimizing patient safety and treatment efficacy. Continued interdisciplinary research and standardized clinical protocols are essential to advance early detection, risk stratification, and tailored interventions, thereby preserving the therapeutic benefits of immunotherapy while mitigating cardiovascular risks.

## 1 Introduction

In recent years, remarkable advancements in immune-based cancer therapies have offered the promise of long-term remission and potential cures for many cancer patients (Maus et al., 2014; Zhang et al., 2015). However, despite these therapeutic breakthroughs, immune-based treatments can cause serious adverse effects, including immune effector cell-associated neurotoxicity syndrome (ICANS) and cytokine release syndrome (CRS), both of which can be fatal (Ghosh et al., 2020; Neelapu et al., 2018). As a result, the advancement and broad implementation of these therapies in clinical settings may be impacted. Notably, cardiovascular toxicities associated with immunotherapy have emerged as a substantial source of morbidity and mortality, limiting the broader implementation of these therapies in clinical practice (Stein-Merlob et al., 2021a; Pareek et al., 2018). Cardiovascular complications can range from mild arrhythmias to severe cardiotoxicity, significantly impacting patient outcomes and quality of life (Lefebvre et al., 2020; Camilli et al., 2023).

**FIGURE 1:**
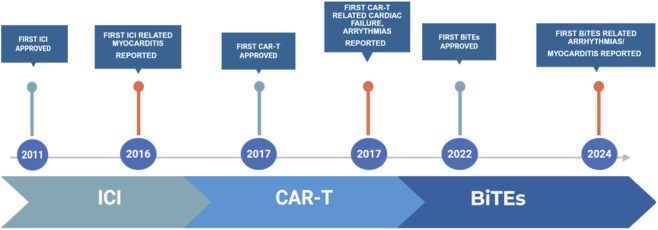
Timeline of immunotherapies and emergence of related cardiotoxicities reported.

Three primary classes of cancer immunotherapies have notably revolutionized clinical practice: ICIs, CAR-T therapies, and bispecific antibodies. Additional immune-based modalities including therapeutic cancer vaccines and oncolytic viruses, particularly in combination with ICIs are also increasingly used and have rare but reported cardiovascular toxicities (Figure 1).

ICIs, such as antibodies targeting programmed cell death-1 (PD-1), programmed cell death ligand-1 (PD-L1), and cytotoxic T-lymphocyte-associated protein 4 (CTLA-4), have significantly improved outcomes across various malignancies (Wojtukiewicz et al., 2021). ICIs function by blocking inhibitory signals that cancer cells exploit to evade immune surveillance, thereby reactivating cytotoxic T-cell mediated anti-tumor responses. Notably, ICIs have dramatically improved prognosis in advanced, non-small cell lung cancer (Olivares-Hernández et al., 2023), renal cell carcinoma (Thouvenin et al., 2023), and several hematological malignancies (Tsumura et al., 2023), establishing them as cornerstones of modern oncology.

CAR-T therapies represent another transformative development, particularly effective in hematologic malignancies like acute lymphoblastic leukemia (ALL), non-Hodgkin lymphoma, and multiple myeloma (Goyco Vera et al., 2024; Zhou et al., 2024). CAR-T therapies involve genetically engineering patients’ own T-cells to express synthetic receptors capable of recognizing and eliminating cancer cells. The remarkable efficacy of CAR-T therapies has led to durable remissions in previously refractory malignancies, significantly changing the therapeutic landscape and prognosis for these patients.

Bispecific antibodies, the third prominent category of cancer immunotherapy, function uniquely by simultaneously binding two distinct antigens, one on cancer cells and another on immune effector cells. This dual targeting facilitates potent immune activation directly at the tumor site, leading to strong anti-tumor activity. Bispecific T-cell engagers (BiTEs), a notable example within this class, have demonstrated impressive efficacy, especially in hematologic cancers (Tian et al., 2021; Lyons and Gore, 2024), further expanding immunotherapeutic options.

Despite the revolutionary advancements and notable clinical successes of these therapies, there is increasing recognition of significant cardiovascular complications associated with their use. These cardiovascular toxicities, collectively termed “cardio-immunotoxicities,” cover a spectrum of clinical manifestations, including myocarditis (Zhou et al., 2025), pericarditis (Kong Y. et al., 2024), arrhythmias (Zhou et al., 2025; Kong Y. et al., 2024), cardiomyopathy (Kong Y. et al., 2024; Joseph et al., 2025), heart failure (Joseph et al., 2025), and acute coronary syndromes, venous thromboembolism and arterial thrombotic events which are increasingly recognized in ICI-treated populations and may reflect immune-driven endothelial dysfunction and accelerated atherosclerotic inflammation (Kong Y. et al., 2024; Efentakis et al., 2025). Such complications can negatively impact patient prognosis and overall survival, potentially reducing the benefits provided by cancer immunotherapies.

The mechanisms underlying cardio-immunotoxicities are complex and multifactorial, often involving immune-mediated inflammation (Du et al., 2025), direct cardiotoxic effects (Baik et al., 2021), endothelial dysfunction (Baik et al., 2021; Efentakis et al., 2025), and CRS (Du et al., 2025), particularly pronounced with CAR-T therapies. The unpredictable nature, rapid onset, and potentially fatal outcomes associated with these cardiovascular events have prompted increased vigilance and significant research efforts directed toward early detection, risk stratification, and management strategies.

Given these developments, this review aims to comprehensively evaluate the current understanding of cardiovascular complications associated with cancer immunotherapies, elucidate the mechanisms underlying cardiotoxicities, highlight the clinical incidence and presentation of these adverse effects, review diagnostic methodologies, and discuss current management strategies. Additionally, the review will address existing gaps in knowledge, emphasize the need for standardized guidelines and protocols, and propose future research directions necessary to optimize patient care.

By integrating existing data and recent advancements, this review aims to provide clinicians, oncologists, cardiologists, and researchers with a cohesive framework to navigate the complexities associated with immunotherapy-induced cardiovascular complications. Such insights are essential for maximizing therapeutic efficacy, minimizing risks, and ultimately improving clinical outcomes for cancer patients undergoing innovative immunotherapeutic interventions.

## 2 Methodological approach

We performed a literature search using PubMed to identify studies on cardiotoxicities associated with immune checkpoint inhibitors, chimeric antigen receptor (CAR) T-cell therapies, and bispecific T-cell engagers/CD3 bispecific antibodies. Search terms included combinations of “immune checkpoint inhibitor,” “PD-1,” “CTLA-4,” “CAR-T,” “bispecific,” “BiTEs,” “cardiotoxicity,” “myocarditis,” “heart failure,” “arrhythmia,” and “cytokine release syndrome,” limited to human, English-language publications. The primary search window covered January 2014 to November 2025, with key reviews and guidelines.

## 3 Types of immunotherapies and their cardiotoxic profiles

### 3.1 Immune checkpoint inhibitors (ICIs)

ICIs have transformed treatment outcomes across a wide range of solid tumors and hematologic malignancies (Kong X. et al., 2024). Approved ICIs now target four major checkpoint pathways: PD-1, PD-L1, CTLA-4, and LAG-3. PD-1 inhibitors include nivolumab, pembrolizumab, cemiplimab, dostarlimab, retifanlimab, toripalimab, and tislelizumab; PD-L1 inhibitors include atezolizumab, durvalumab, avelumab, and cosibelimab; CTLA-4 inhibitors include ipilimumab and tremelimumab; and the first-in-class LAG-3 inhibitor relatlimab is approved as a fixed-dose combination with nivolumab (Arafat Hossain, 2024).

Lymphocyte-activation gene-3 (LAG-3) is a co-inhibitory receptor expressed on activated T-cells, with therapeutic synergy when blocked alongside PD-1. LAG-3 inhibition entered routine practice following regulatory approvals of nivolumab/relatlimab for unresectable or metastatic melanoma (2022) as it has demonstrated clinical benefit in melanoma; however, accumulating safety data suggests a higher incidence of myocarditis and multisystem immune-related adverse events (irAEs) compared with PD-1 monotherapy (Stefanou et al., 2025). Although absolute rates remain low, this pattern mirrors prior observations with dual PD-1 + CTLA-4 inhibition, suggesting that layered checkpoint blockade can amplify autoimmune potential and erode peripheral tolerance in cardiac tissue. Rare cases of clinically significant myocarditis, myositis, and conduction abnormalities have been reported, typically early in the treatment course and often in combination regimens (Stefanou et al., 2025). Because LAG-3 inhibition is now part of standard ICI practice, cardiovascular immune-related adverse events (irAEs) should be considered within the broader ICI cardiotoxicity framework, particularly in combination regimens (Tawbi et al., 2022).

These agents are used as monotherapy or, increasingly, in combination regimens that enhance anti-tumor efficacy but also amplify immune-related toxicities (Liu et al., 2021). To date, the FDA has approved 10+ checkpoint antibodies across PD-1, PD-L1, CTLA-4, and LAG-3 targets, with multiple agents per class and expanding indications across solid and hematologic malignancies (Vaddepally et al., 2020). As ICIs become standard components of first-line cancer therapy, recognition of their cardiovascular immune-related adverse events (irAEs) has become critical for oncologists and cardiology teams worldwide. It is commonly recommended that ICI treatment be discontinued when patients experience severe cardiotoxicity, with the treatment plan reconsidered only after the cardiac condition has stabilized (Becker, 2025). However, whether it is advisable to re-challenge patients with the same regimen remains controversial. Many cancer patients with cardiovascular diseases consequently receive conservative treatment, which may result in poorer clinical outcomes.

#### 3.1.1 Reported cardiotoxicities, epidemiology and risk factors

Although initially considered rare, contemporary datasets suggest that myocarditis occurs in approximately 0.6%–1.1% of patients receiving ICIs, with higher frequencies observed when PD-1/PD-L1 inhibitors are combined with CTLA-4 therapy. A landmark multicenter registry reported that combination therapy increases the risk of myocarditis five-fold compared with PD-1 monotherapy, with median onset typically after 2–3 doses (Mahmood et al., 2018; Wang et al., 2018). Meta-analyses of randomized trials also demonstrate elevated risks of myocarditis, pericardial diseases, and possibly arrhythmias in ICI-treated cohorts (Moslehi et al., 2018). Specific risk factors include combination therapy (PD-1/PD-L1 plus CTLA-4), pre-existing autoimmune disease, concurrent multi-organ irAEs (particularly when myositis or myasthenia gravis are present), older age, and possibly underlying cardiac disease (with emerging data) (Hu et al., 2019). Because clinical trials often under-report rare irAEs, the real-world incidence may be modestly higher than early estimates (Escudier et al., 2017).

ICI-related cardiotoxicity comprises a broad spectrum of clinical syndromes. Myocarditis remains the most feared toxicity due to its potential for rapid progression and high mortality. Early reports from pharmacovigilance databases and prospective registries describe myocarditis typically occurring within the first 4–6 weeks of therapy and presenting with fulminant features, arrhythmias, and cardiogenic shock. Fatality rates in early cohorts exceeded 40%–50%, particularly in combination PD-1/CTLA-4 regimens (Johnson et al., 2016; Houmsse et al., 2025). Beyond myocarditis, ICIs have been associated with pericarditis, pericardial effusion, atrial and ventricular arrhythmias, conduction abnormalities, and stress-induced (Takotsubo-like) cardiomyopathy (Palaskas et al., 2020; Varricchi et al., 2018); including venous thromboembolism (VTE) and arterial thromboembolism (ATE). Large observational cohorts and systematic analyses suggest clinically meaningful rates of VTE/ATE during ICI therapy, and mechanistic work supports immune-mediated endothelial activation and plaque inflammation as plausible contributors (Khorana et al., 2023). Additional presentations include acute coronary syndromes, possibly mediated by accelerated inflammation in atherosclerotic plaques, and vasculitis syndromes involving coronary and peripheral vessels. Heart failure with reduced ejection fraction, either secondary to myocarditis or as an independent phenotype of myocardial inflammation, has also been documented.

#### 3.1.2 Proposed mechanisms of ICI-associated cardiotoxicity

**FIGURE 2:**
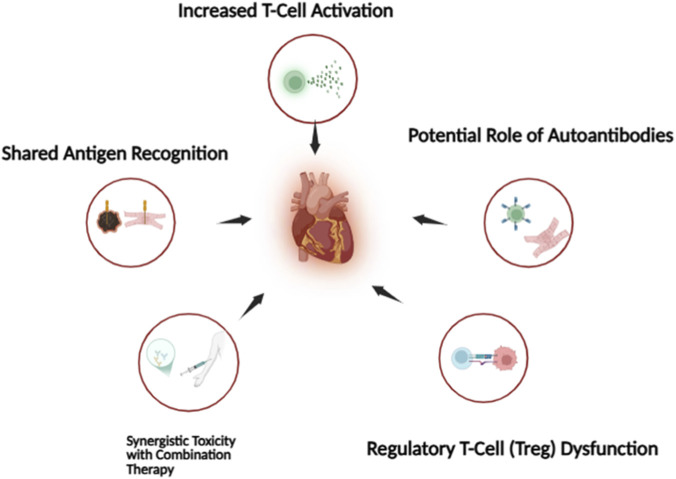
Proposed mechanisms of ICI-associated cardiotoxicity.

ICIs regenerate anti-tumor immunity by targeting inhibitory pathways such as PD-1, PD-L1, and CTLA-4. While these pathways are crucial for preventing tumor immune evasion, they are also central to maintaining peripheral immune tolerance. Their disruption can lead to a spectrum of immune-related adverse events (irAEs), with myocarditis being among the most severe and fatal, carrying mortality rates as high as 40%–50% in reported cohorts (Salem et al., 2018)^,^. Multiple convergent mechanisms drive ICI-associated cardiotoxicity, with evidence pointing to dysregulated T-cell activity, shared antigen cross-reactivity, and innate immune amplification (Figure 2).

*Loss of Peripheral Tolerance and Increased T-Cell Activation*: PD-1/PD-L1 and CTLA-4 signaling normally limit excessive immune activation and protect healthy tissues from autoimmunity. ICIs remove these inhibitory signals, enabling unchecked expansion and effector activity of cytotoxic T-cells. Preclinical studies support a cardioprotective role for PD-1/PD-L1 signaling, but the cardiac phenotype in PD-1 deficient mice is strain and context-dependent rather than uniformly myocarditic (Nishimura et al., 1999). In humans, early biopsies from ICI-myocarditis patients consistently show CD8^+^-dominant lymphocytic infiltration with associated myocyte necrosis, a hallmark of T-cell mediated myocardial injury (Palaskas et al., 2020).

*Molecular Mimicry and Shared Antigen Recognition*: One of the most compelling mechanistics is the discovery of identical T-cell receptor (TCR) clonotypes infiltrating both tumors and the myocardium (Johnson et al., 2016). This strongly suggests antigen cross-reactivity where T-cells activated against tumor proteins also recognize homologous cardiac antigens. Melanoma patients may be particularly predisposed, as melanocyte and cardiomyocyte proteins share conserved sequences, promoting “on-target, off-tumor” autoimmune injury (Linette et al., 2013).

*Synergistic Toxicity with Combination Therapy*: Combination therapy (e.g., nivolumab + ipilimumab) significantly increases myocarditis risk up to 4–6 times higher than monotherapy. Mechanistically: CTLA-4 blockade enhances T-cell priming in lymph nodes. PD-1 blockade amplifies effector T-cell function in peripheral tissues. Together, these effects create a potent, synergistic immune environment that accelerates myocardial infiltration, inflammation, and tissue destruction (Hu et al., 2019).

*Cytokine-Mediated Injury and Immune Amplification*: Hyperactivated T-cells release inflammatory cytokines including IFN-γ, TNF-α, IL-1β, and IL-6. These cytokines promote myocyte apoptosis, increase expression of MHC molecules on cardiomyocytes, damage the microvascular endothelium, recruit macrophages and neutrophils that further amplify inflammation. Some severe cases exhibit features overlapping with cytokine-release syndrome, supporting a systemic inflammatory contribution (Zheng et al., 2025).

*Direct Myocardial Immune Cell Infiltration*: Histopathology from fatal cases shows dense CD8^+^ T-cell infiltration, activated macrophages with high expression of CD-68, scattered or diffuse myocyte necrosis, minimal fibrosis early in disease. Cardiomyocytes upregulate PD-L1 during stress as a protective counter-regulatory mechanism, but PD-1/PD-L1 blockade nullifies this defense, rendering the myocardium uniquely vulnerable to cytotoxic attack (Ostos-Mendoza et al., 2025; Jimenez et al., 2023; Sobol et al., 2020; Wing et al., 2008).

*Regulatory T-Cell (Treg) Dysfunction*: CTLA-4 is highly expressed on Tregs, where it is essential for suppressive function (Wing et al., 2008). Blockade of CTLA-4 reduces Treg activity, removes an additional layer of peripheral tolerance, and permits expansion of autoreactive T-cell clones (Liu et al., 2016; Klocke et al., 2016). Mice with Treg depletion rapidly develop myocarditis that mirrors the pathology of ICI-treated patients (Klocke et al., 2016).

*Potential Role of Autoantibodies*: Although T-cell mechanisms dominate, several reports describe autoantibodies targeting cardiac antigens (e.g., troponin I, β-1 adrenergic receptors) (Jahns et al., 2004). Whether these antibodies are primary mediators of injury or secondary to myocardial necrosis remains unclear, but they may amplify tissue damage or contribute to late-phase cardiac dysfunction.

#### 3.1.3 Diagnosis and recommended monitoring

Contemporary international cardio-oncology guidelines recommend structured baseline assessment and early-cycle monitoring. Baseline ECG, high-sensitivity cardiac troponin, and natriuretic peptides should be obtained prior to ICI initiation. Serial troponin measurements during the first 4–6 treatment cycles are advised for higher-risk patients (e.g., combination therapy, prior cardiovascular disease). Baseline echocardiography is reasonable in patients with cardiac risk factors (Raschi et al., 2023). When myocarditis is suspected, elevated troponin coupled with new ECG abnormalities warrants urgent echocardiography and cardiac MRI using the updated Lake Louise Criteria. Cardiac MRI provides excellent tissue characterization and can reveal edema, late gadolinium enhancement, and supportive markers of myocardial inflammation (Murtagh et al., 2024; Patel et al., 2025). Endomyocardial biopsy remains the definitive diagnostic standard when noninvasive studies are inconclusive or when precise histopathologic diagnosis is required for management decisions.

#### 3.1.4 Management strategies

Prompt recognition and immediate treatment are essential. Discontinuation of the ICI and initiation of high-dose corticosteroids (e.g., methylprednisolone 1–2 mg/kg/day or pulse dosing in severe cases) form the cornerstone of therapy (Mahmood et al., 2018). Evidence suggests that earlier steroid initiation and higher initial dosing reduce major adverse cardiac events (MACE) (Zhang et al., 2020). Patients with hemodynamic compromise should be managed in intensive care units with mechanical circulatory support as needed. For steroid-refractory myocarditis, several immunomodulatory agents have been used, including Mycophenolate mofetil, Intravenous immunoglobulin (IVIG), Anti-thymocyte globulin, abatacept, a CTLA-4 agonist that restores inhibitory signaling (supported by case series and ongoing phase II trials) (Salem et al., 2019). Rechallenge with ICIs after a myocarditis episode is highly individualized and generally discouraged in moderate to severe cases due to risk of recurrence (Stein-Merlob et al., 2021b) (Table 1).

**TABLE 1:** Comparative clinical and mechanistic profiles of major cardiotoxicities.

Feature	Immune checkpoint inhibitors (ICIs)	CAR T-cell therapy	BiTEs/CD-3 bispecific antibodies
Core mechanism	Releases immune “brakes” by blocking PD-1/PD-L1, CTLA-4 (and LAG-3 via nivolumab/relatlimab)	Engineered patient T-cells recognize a tumor antigen (e.g., CD-19/BCMA) and kill tumor cells	Antibody bridges CD-3 (T-cell) to tumor antigen, triggering T-cell killing
Typical cancer settings	Broad solid tumors + some hematologic malignancies	Mostly hematologic malignancies	Mostly hematologic malignancies (increasingly earlier lines)
Signature CV toxicities	Myocarditis (highest concern); arrhythmias/conduction disease; pericarditis/effusion; cardiomyopathy/HF; ACS; VTE/ATE	CRS-linked: hypotension/vasoplegia; SVT/AF; transient LV dysfunction; rare shock/cardiac arrest	CRS-linked: hypotension; tachyarrhythmias; LV dysfunction; vasoplegia; thromboembolism; rare myocarditis/QT issues
Dominant pathobiology	Immune loss of tolerance → T-cell predominant myocardial inflammation (often early)	Cytokine-driven myocardial depression + endothelial dysfunction during CRS	Similar to CAR-T: cytokine surge + endothelial dysfunction during CRS
Typical timing	Often early (first 4–8 weeks), but variable	Days-3 weeks (CRS window)	Step-up dosing period, usually first 1–2 weeks
Suggested baseline checks	ECG + hs-troponin (±BNP/NT-proBNP); echo if risk factors	ECG + echo (±strain) + hs-troponin + BNP/NT-proBNP	ECG + hs-troponin (±BNP/NT-proBNP); echo if high risk
Trigger-based workup	Rising troponin and/or new ECG changes → echo → CMR if stable; consider biopsy if uncertain	Symptoms/biomarker rise during CRS → echo; CMR if stable and myocarditis question persists	Same as CAR-T during CRS/step-up dosing
First-line management	Hold ICI + high-dose steroids; escalate immunosuppression if refractory; ICU if unstable	Treat CRS (tocilizumab ± steroids) + hemodynamic/arrhythmia support	Treat CRS (tocilizumab ± steroids) + supportive cardiac care
Rechallenge (general)	Usually avoid after moderate–severe myocarditis; individualized in mild fully resolved cases	Consider only after full recovery; multidisciplinary decision	Consider only after full recovery; multidisciplinary decision

#### 3.1.5 Gaps in knowledge and future directions

Despite rapid advances, substantial gaps remain. Precise incidence estimates stratified by ICI agent, tumor type, and combination regimen are lacking. There are no validated risk-prediction tools, and screening protocols (frequency of troponin testing, role of natriuretic peptides) vary widely across centers and guidelines. Optimal steroid taper duration and standardized pathways for second-line immunosuppression remain unknown, as data derive largely from case series. Prospective clinical trials evaluating targeted therapies such as abatacept, JAK inhibitors, or IL-6 blockade are ongoing and will be crucial to establishing evidence-based regimens.

As the use of ICIs continues to expand, coordinated research efforts and harmonized clinical guidelines are needed to improve early detection, risk stratification, and outcomes in patients experiencing cardiovascular irAEs.

### 3.2 CAR-T cell therapy

CAR-T therapy represents one of the most transformative advances in modern oncology. These living drugs are engineered from autologous T-cells to recognize and eliminate malignant B-cells or plasma cells, resulting in high response rates even among heavily pretreated patients (An et al., 2025). As of 2025, six CAR-T products have gained FDA approval, targeting CD-19 (tisagenlecleucel, axicabtagene ciloleucel, lisocabtagene maraleucel, brexucabtagene autoleucel) and BCMA (idecabtagene vicleucel, ciltacabtagene autoleucel) for diseases spanning B-ALL, LBCL, MCL, FL, CLL/SLL, and multiple myeloma (Jangavali et al., 2025).

While the anti-tumor efficacy is remarkable, the profound immune activation they induce, often necessary for therapeutic effects, can precipitate significant systemic toxicities. Cardiovascular adverse events (CVAEs) are increasingly recognized as clinically important complications, typically occurring in the context of CRS or neurotoxicity (ICANS). Growing experience has highlighted the need for proactive cardiovascular surveillance and multidisciplinary care.

#### 3.2.1 Reported cardiotoxicities, epidemiology and risk factors

Although relatively uncommon compared with CRS and neurotoxicity, CVAEs remain significant due to their morbidity. Across trials and real-world cohorts, reported incidents were as follows:

Hypotension requiring vasopressors occurs in up to 30%–40% of patients during CRS (grade ≥2 CRS) (Simbaqueba et al., 2020). Supraventricular arrhythmias were seen in 5%–12% of patients, including atrial fibrillation and atrial flutter. LV systolic dysfunction was reported in 5%–10% of patients, often reversible with CRS resolution. Ventricular arrhythmias and cardiac arrest are rare but associated with high mortality (Koeckerling et al., 2024).

Evidence from multicenter cohorts and registries identifies several consistent predictors like Higher CRS grade (most powerful predictor of cardiotoxicity), Elevated IL-6, CRP, ferritin, and troponin, Older age (≥60), Baseline cardiovascular disease (coronary artery disease, HF, hypertension), Prior anthracycline exposure, chest radiation, or autologous stem-cell transplant, High tumor burden and greater *in vivo* CAR-T expansion kinetics. Most CV events occur within the first 14–21 days post-infusion, closely paralleling CRS onset and peak cytokine elevation (Camilli et al., 2023; Simbaqueba et al., 2020; Kotch et al., 2019).

#### 3.2.2 Proposed mechanisms of associated cardiotoxicity

Cardiovascular injury after CAR-T therapy is largely indirect, mediated through profound immune activation rather than direct myocardial cytotoxicity. Several interconnected biological mechanisms have been proposed:

*Cytokine-Mediated Myocardial Depression*: CRS triggers explosive release of inflammatory cytokines including IL-6, IL-1β, IFN-γ, GM-CSF, and TNF-α which exert widespread hemodynamic and myocardial effects. IL-6, the central mediator of CRS, induces nitric oxide synthase expression, causing vasoplegia and depressed myocardial contractility resembling septic cardiomyopathy (Camilli et al., 2023; Simbaqueba et al., 2020). IFN-γ and TNF-α contribute to mitochondrial dysfunction, impairing ATP production in cardiomyocytes. High cytokine burden is associated with capillary leak, interstitial edema, and impaired oxygen delivery. Functional myocardial depression is often reversible, reflecting the transient nature of inflammatory injury.

*Endothelial/Microvascular Dysfunction*: Endothelial activation represents a critical upstream event in CRS pathophysiology (Zhang et al., 2024). Preclinical and clinical studies demonstrated elevated angiopoietin-2, von-Willebrand factor (vWF), and endothelial adhesion molecules during CRS (Pinto and Krenciute, 2024). Disruption of endothelial tight junctions causing capillary leak syndrome, third-spacing, intravascular volume depletion, and reflex tachycardia. Microvascular dysfunction contributes to hypotension, organ hypoperfusion, and possible cardiac ischemia independent of coronary obstruction. Emerging data suggest that endothelial injury is a common nexus for CAR-T’s major toxicities: the same endothelial perturbation that increases BBB permeability in ICANS and triggers coagulopathy in CRS may underlie stress cardiomyopathy and arrhythmias seen in these patients. This has generated interest in adjunctive therapies targeting the endothelium to mitigate toxicity.

*Rare On-Target/Off-Tumor Toxicity*: While CD-19 and BCMA are not known to be expressed in myocardial cells, rare case reports suggest accidental recognition of low-level or cross-reactive epitopes and expansion of autoreactive clones during massive immune activation (Camilli et al., 2023). These remain theoretical and poorly substantiated clinically but highlight the need for ongoing mechanistic research.

*Biomarkers Supporting Mechanistic Links*: Multiple studies documents that levels of CRP, ferritin, IL-6, and cardiac troponins track closely with CRS severity, cardiovascular dysfunction, and mortality risk. Troponin elevation often precedes LV dysfunction and predicts hemodynamic instability. Ferritin >10,000 ng/mL is associated with severe CRS and high vasopressor requirement (Simbaqueba et al., 2020; Kotch et al., 2019).

#### 3.2.3 Diagnosis and recommended monitoring

Given the predictable early physiological window during which cardiovascular events occur after CAR-T infusion, current guidelines emphasize the importance of proactive baseline assessment and vigilant monitoring during the high-risk period. A comprehensive baseline cardiovascular evaluation is essential for identifying patients at increased risk and typically includes a 12-lead ECG, a transthoracic echocardiogram to measure left ventricular ejection fraction and myocardial strain when available, and baseline measurements of high-sensitivity troponin and BNP or NT-pro BNP. This assessment should also include a thorough review of prior cardiotoxic therapies, such as anthracyclines or chest radiation, and an evaluation of traditional cardiovascular risk factors (Lyon et al., 2022). Patients with known left ventricular dysfunction or significant cardiac history should undergo cardio-oncology consultation prior to receiving lymphodepleting chemotherapy to ensure individualized risk stratification and optimization of care (Hu et al., 2021).

During the early post-infusion period when CRS is most likely to occur, intensive monitoring is essential. Continuous telemetry is recommended to detect arrhythmias, which are common in the context of fever, electrolyte disturbances, and systemic inflammation (Neelapu et al., 2018). High-risk patients may benefit from daily troponin measurements and serial inflammatory markers, as early rises in troponin have been shown to correlate with impending hemodynamic instability and left ventricular dysfunction. Meticulous fluid management is particularly important to mitigate capillary leak, a hallmark of CRS physiology, while clinicians should avoid QT-prolonging medications whenever possible due to the compounded arrhythmic risk. This period of close observation typically spans the first two to 3 weeks following CAR-T infusion, aligning with the window of peak cytokine activity (Camilli et al., 2023; Hu et al., 2021).

When symptoms (such as dyspnea, chest discomfort, or palpitations) or biomarker abnormalities raise concern for cardiac injury, targeted diagnostic evaluation is warranted. Repeating transthoracic echocardiography is the first step, allowing for assessment of left ventricular ejection fraction, wall motion patterns, and the presence of pericardial effusion. When clinically feasible and the patient is hemodynamically stable, cardiac MRI can provide additional clarity by distinguishing between inflammatory myocarditis, ischemic injury, or stress-induced cardiomyopathy. Although ischemic evaluation should be pursued when clinically indicated, true obstructive coronary events are relatively uncommon in this setting. Diagnostic complexity often arises because CRS-driven cytokine-mediated myocardial injury can mimic myocarditis both clinically and biochemically; thus, differentiating these entities remains an active and evolving area of investigation in cardio-oncology (Simbaqueba et al., 2020; Dalal et al., 2022; Ottawa ON: Canadian Agency for Drugs and Technologies in Health, 2024).

#### 3.2.4 Management strategies

Management of CAR-T–associated cardiovascular complications centers on controlling the underlying inflammatory driver cytokine release syndrome (CRS) while simultaneously providing cardiology-directed supportive care. Because CRS physiology accounts for the majority of cardiovascular events, timely recognition and intervention are essential. Current ASTCT guidelines form the backbone of CRS treatment. For Grade 1 CRS, supportive care with antipyretics and intravenous fluids is generally sufficient. In patients with Grade ≥2 CRS, anti IL-6 therapy with tocilizumab is the first-line intervention and often produces rapid improvement in hypotension, tachycardia, and fever. Corticosteroids, such as dexamethasone, are added for persistent symptoms or early signs of ICANS to temper the inflammatory response (Lee et al., 2019; Santomasso et al., 2021; Maus et al., 2020). For refractory CRS, escalation of immunomodulatory therapy is required and may include additional IL-6 blockade with siltuximab or IL-1 inhibition with anakinra, especially in severe inflammatory phenotypes. In the most critical cases, patients may require aggressive ICU-level care, including vasopressors, high-flow oxygen or ventilatory support, and careful hemodynamic monitoring. Prompt cytokine-directed therapy not only stabilizes systemic inflammation but can also prevent progression to acute cardiac dysfunction (Giavridis et al., 2018; Diorio et al., 2022).

Alongside CRS treatment, cardiac-specific management follows established cardiology principles adapted for the unique constraints of CAR-T therapy. Arrhythmias should be managed by correcting electrolyte abnormalities, using rate-control agents such as β-blockers when tolerated, and pursuing rhythm-control strategies when clinically indicated. Clinicians should minimize or avoid QT-prolonging medications, which are commonly used in oncology settings and may increase arrhythmic risk. Heart failure management includes judicious diuresis to avoid exacerbating pulmonary edema due to capillary leak, with initiation of guideline-directed medical therapy once the acute CRS phase has resolved and if LV dysfunction persists. In cases of cardiogenic shock, temporary inotropic support may be required. For circulatory support, norepinephrine is the preferred first-line vasopressor for CRS-related vasoplegia, with vasopressin added in cases of refractory hypotension. Mechanical circulatory support (such as ECMO or Impella) is rarely needed but may be considered for life-threatening cardiogenic shock unresponsive to medical therapy (Ganatra et al., 2019).

Following stabilization, decisions regarding ongoing CAR-T monitoring, the safety of additional cell-infusion cycles, and eligibility for future immunotherapies must be individualized. These determinations require close collaboration between cardio-oncology, hematology, and intensive care teams to balance oncologic benefit, treatment response, and risk of recurrent cardiovascular complications (Table 1).

#### 3.2.5 Gaps in knowledge and future directions

Despite meaningful advances in understanding CAR-T–associated cardiotoxicity, numerous gaps in knowledge remain and continue to limit the development of standardized care pathways. One major challenge is the lack of uniform surveillance protocols: the optimal timing and frequency of troponin assessment, natriuretic peptide measurement, and echocardiographic monitoring are not well defined, and existing practices vary widely across institutions. Similarly, there is an urgent need for validated predictive models capable of identifying patients at highest risk for cardiovascular events. Current approaches rely largely on single biomarkers such as IL-6, ferritin, or troponin, but a more robust, multivariable risk score incorporating CRS kinetics, CAR-T expansion patterns, baseline tumor burden, and patient comorbidity would more accurately stratify risk and guide individualized monitoring. Another critical uncertainty involves the true incidence of myocarditis and pericarditis. Because the clinical features of CRS-driven inflammatory cardiac injury overlap with myocarditis, these conditions are likely underdiagnosed, highlighting the importance of prospective registries with standardized outcome adjudication.

Long-term cardiovascular outcomes also remain poorly understood; the trajectory of left ventricular recovery, late arrhythmic risk, and the possibility of persistent microvascular injury have not been well characterized, underscoring the need for survivorship studies particularly as CAR-T is increasingly used in earlier lines of therapy and in younger patient populations. Finally, there is limited evidence on the comparative effectiveness of CRS-directed versus cardiac-directed interventions. Optimal timing for therapies such as tocilizumab, anakinra, or corticosteroids relative to the onset of cardiac injury remains uncertain, and it is not clear when escalation of direct cardiac support should supersede intensified CRS management. Addressing these gaps through prospective, harmonized research efforts will be essential to refining clinical pathways and improving cardiovascular outcomes in CAR-T recipients.

### 3.3 Bispecific T-Cell engagers (BiTEs) and CD-3 bispecific antibodies

Bispecific T-cell engagers (BiTEs) and related CD-3-bispecific antibodies represent a rapidly expanding class of immunotherapies designed to redirect endogenous cytotoxic T-cells toward malignant targets. These engineered molecules contain dual antigen-binding domains one recognizing a tumor-associated antigen and the other binding CD-3 on T-cells thereby inducing immune synapse formation and dose-dependent T-cell activation (Devasia et al., 2024). Clinically approved examples include blinatumomab (CD-19×CD-3) for B-cell acute lymphoblastic leukemia (Goebeler and Bargou, 2016), teclistamab (BCMA × CD-3) and talquetamab (GPRC5D × CD-3) for relapsed or refractory multiple myeloma (Devasia et al., 2024). Numerous additional CD-3-bispecifics, including CD-20×CD-3 monoclonal antibodies (epcoritamab, mosunetuzumab), are advancing through clinical development and real-world integration (Crombie et al., 2024). By bypassing MHC restrictions and leveraging the patient’s endogenous T-cell population, these agents offer potent anti-tumor activity even in heavily pretreated populations (Sayed et al., 2024).

#### 3.3.1 Reported cardiotoxicities, epidemiology and risk factors

Although BiTE-related cardiovascular adverse events (CVAEs) occur less frequently than hematologic, neurologic, or infectious complications, accumulating pharmacovigilance analyses, regulatory databases, and cohort studies highlight a non-negligible cardiotoxicity profile. Reported CVAEs include sinus tachycardia, atrial and ventricular arrhythmias, hypotension, vasoplegic shock, left ventricular (LV) dysfunction/heart failure, thromboembolic events, and less commonly myocarditis and QT-interval prolongation (Shan C. et al., 2025).

Signal analyses from the FDA Adverse Event Reporting System (FAERS) reveal that teclistamab shows a notable disproportionality signal for myocarditis and QT prolongation, suggesting either heightened susceptibility or more comprehensive reporting within multiple myeloma cohorts. In contrast, blinatumomab demonstrates a more prominent signal for thromboembolic phenomena, likely influenced by cytokine-mediated endothelial activation, concurrent steroid exposure, and the prothrombotic milieu of acute leukemia.

Although the overall proportion of CVAEs within the broader BiTE toxicity landscape remains modest, typically small relative to CRS and neurotoxicity, their clinical impact is significant due to the potential for hemodynamic instability, arrhythmia-related morbidity, or progression to overt heart failure. As BiTE prescribing increases and use expands into earlier disease settings, systematic cardiovascular surveillance will become more critical.

Meta-analytic assessments of FAERS data suggest that approximately 8%–20% of BiTE-associated adverse event reports include cardiovascular complications, although this likely underestimates the true incidence due to variable reporting and lack of systematic monitoring. Several consistent epidemiologic patterns have emerged. Cardiovascular events tend to occur more frequently during the early step-up dosing phase, when the risk and severity of cytokine release syndrome (CRS) are greatest. Real-world cohorts of patients with multiple myeloma treated with teclistamab demonstrate that most CRS episodes are grade 1–2, with serious cardiovascular events remaining relatively uncommon; however, the absence of routine prospective cardiology screening in these studies limits the ability to quantify risk precisely. Across datasets, higher cardiovascular vulnerability appears concentrated in patients with preexisting cardiovascular disease, high tumor burden, active infections or inflammatory states, or prior exposure to cardiotoxic therapies such as anthracyclines or proteasome inhibitors. Additionally, older age and the presence of metabolic comorbidities further elevate susceptibility to BiTE-associated cardiovascular adverse events, emphasizing the need for tailored monitoring in high-risk populations (Sayed et al., 2024; Graf et al., 2024; Grunblatt and Akhter, 2025).

#### 3.3.2 Proposed mechanisms of associated cardiotoxicity

The mechanistic underpinnings of BiTE-associated cardiotoxicity closely parallel those observed with CAR-T therapy and other potent T-cell activating immunotherapies. A central driver is CD-3-mediated cytokine release, in which BiTE engagement triggers rapid T-cell activation and proliferation accompanied by secretion of proinflammatory mediators such as IL-6, IFN-γ, TNF-α, and GM-CSF. These cytokine surges can precipitate tachyarrhythmias and conduction abnormalities, induce systemic vasodilation and hypotension, cause sepsis-like myocardial depression through nitric oxide–dependent pathways, and promote capillary leak with resultant fluid shifts and hemodynamic instability. In parallel, widespread immune activation contributes to endothelial dysfunction, characterized by upregulation of adhesion molecules, increased vascular permeability, and activation of coagulation pathways. This endothelial injury can manifest clinically as hypotension, edema and third-spacing, microvascular perfusion defects, and thromboembolic events (Simbaqueba et al., 2020; Ganatra et al., 2022).

Although less common, a third mechanistic category involves on-target/off-tumor toxicity, wherein BiTEs may inadvertently engage antigens expressed at low levels on cardiac tissue. While this risk appears minimal with current targets such as BCMA, GPRC5D, and CD-19, it remains a theoretical concern as newer antigens and multi-target bispecific formats are developed. Together, these pathways highlight the complex interplay between immune activation, vascular biology, and myocardial vulnerability in BiTE-associated cardiotoxicity (Devasia et al., 2024).

#### 3.3.3 Diagnosis and recommended monitoring

Given the predictable window of early toxicity during the step-up dosing phase of BiTE therapy, structured cardiovascular monitoring is essential to ensure timely detection and management of adverse events. A thorough baseline assessment should be performed prior to treatment, including a 12-lead ECG, measurement of troponin and, in higher-risk individuals, natriuretic peptides, as well as a review of any prior exposure to cardiotoxic therapies. During active therapy, patients should undergo continuous telemetry while hospitalized to allow early identification of tachyarrhythmias, hypotension, or rising cardiac biomarkers that may herald cytokine-driven cardiovascular stress. If new symptoms such as dyspnea, chest discomfort, or lightheadedness develop or if biomarkers become abnormal, a symptom-triggered diagnostic workup is warranted. This evaluation typically includes transthoracic echocardiography to assess ventricular function and hemodynamics, with cardiac MRI considered once the patient is clinically stabilized in cases where myocarditis is suspected or when diagnostic clarification is needed. This tiered approach helps capture both overt and subclinical cardiotoxicity during the highest-risk phase of therapy (Sayed et al., 2024; Grunblatt and Akhter, 2025).

#### 3.3.4 Management strategies

The cornerstone of managing cardiovascular toxicity associated with BiTE therapy is prompt treatment of the underlying inflammatory driver cytokine release syndrome (CRS) following ASTCT-aligned therapeutic pathways. Tocilizumab remains the first-line agent for CRS-mediated hemodynamic instability and is frequently combined with corticosteroids when symptoms persist or escalate, or when neurotoxicity coexists. Alongside CRS-directed therapy, clinicians should implement standard guideline-directed treatments for arrhythmia and heart failure, carefully tailoring these interventions to the patient’s hemodynamic profile, comorbid conditions, and the dynamic physiological changes associated with CRS. During any significant cardiovascular adverse event, BiTE therapy should be temporarily held, and decisions regarding rechallenge should be made on an individualized basis, weighing recovery trajectory, underlying disease burden, future treatment options, and multidisciplinary input from cardio-oncology and hematology teams. Recently issued expert recommendations for CD-20×CD-3 bispecific antibodies have provided detailed and practical toxicity-management algorithms and given their mechanistic overlap with other CD-3 bispecifics, these frameworks are increasingly being adopted as generalizable models for the broader BiTE class (Crombie et al., 2024; Ganatra et al., 2022) (Table 1).

#### 3.3.5 Gaps in knowledge and future directions

Despite growing clinical experience, several important uncertainties persist. The true incidence of BiTE-associated cardiovascular adverse events remains unknown due to the absence of prospective studies with systematically adjudicated cardiac endpoints. Similarly, optimal screening strategies such as the appropriate frequency of biomarker monitoring and clear thresholds for obtaining echocardiography have yet to be defined. Mechanistically, distinguishing cytokine-mediated myocardial depression from true immune-mediated myocarditis remains challenging and requires further translational investigation. In addition, there is a pressing need for comparative-effectiveness studies to determine when CRS-directed treatment alone is adequate versus when escalation to targeted cardiac therapies is warranted. Addressing these gaps will be essential to improving risk stratification and refining cardio-oncology care pathways for patients receiving BiTEs.

### 3.4 Cancer vaccines and oncolytic viruses

Therapeutic cancer vaccines and oncolytic viruses (OVs) represent distinct immunotherapy modalities that prime or amplify host immunity through antigen presentation, dendritic cell activation, and immunogenic tumor cell death. Examples include sipuleucel-T, intravesical BCG, viral vector–based vaccines and clinically used oncolytic viruses such as talimogene laherparepvec (T-VEC) (Heem et al., 2024).

#### 3.4.1 Reported cardiotoxicities, epidemiology and risk factors

Cardiac toxicities associated with cancer vaccines and oncolytic viruses (OVs) are uncommon but increasingly recognized, particularly as these therapies are incorporated into combination immunotherapy regimens (Heem et al., 2024). Among therapeutic vaccines, sipuleucel-T has been linked to isolated cases of inflammatory cardiomyopathy (Heem et al., 2024), while intravesical BCG can, in rare instances especially with systemic BCG cause granulomatous myocarditis presenting with conduction abnormalities, ventricular arrhythmias, or even sudden cardiac death (Geetha et al., 2024). The risk of myocarditis appears further elevated when vaccines are combined with immune checkpoint inhibitors, although the ICI component is typically the dominant driver of cardiotoxicity. Oncolytic viruses, including T-VEC, generally exhibit a favorable cardiac safety profile, with clinical trials and real-world series showing primarily mild, grade 1–2 constitutional symptoms such as self-limiting rashes. Reports of myocarditis are rare and most often arise in the context of combination OV + PD-1 blockade, rather than OV monotherapy. Modern adenoviral and vaccinia-based platforms have deliberately incorporated genetic modifications to minimize native viral cardiotropism, further reducing the likelihood of direct myocardial involvement. Overall, cardiotoxic events with vaccines and OVs remain infrequent; however, risk increases in the setting of combination regimens, preexisting cardiovascular disease, and high systemic inflammatory burden or infection. Consistent with this, published T-VEC studies demonstrate minimal cardiac immune toxicity when used alone, supporting its generally favorable cardiovascular safety in monotherapy settings (Minev et al., 2019).

#### 3.4.2 Proposed mechanisms of associated cardiotoxicity

The proposed mechanisms of cardiotoxicity associated with cancer vaccines and oncolytic viruses vary depending on the specific platform but generally relate to immune activation and tissue-specific host responses. Immune stimulation from these therapies can generate cytokine surges capable of provoking myocardial inflammation or precipitating stress cardiomyopathy, while BCG therapy may trigger delayed-type hypersensitivity reactions or lead to granulomatous infiltration of cardiac tissue in cases of systemic dissemination. Additionally, theoretical concerns arise from viral backbone tropism, as certain wild-type viruses such as coxsackievirus are naturally cardiotropic, although modern engineered oncolytic viral platforms are deliberately modified to minimize these properties. When symptoms suggest possible cardiac involvement, clinicians should apply established pathways for immune-related myocarditis, including prompt evaluation with ECG and troponin testing, transthoracic echocardiography for structural assessment, and cardiac MRI using Lake Louise criteria when myocarditis is suspected and the patient is clinically stable (Murtagh et al., 2024).

#### 3.4.3 Diagnosis and recommended monitoring

Given the generally low but non-zero risk of vaccine- and OV-associated cardiotoxicity, a pragmatic, risk-adapted diagnostic strategy is recommended rather than routine universal screening. For most patients treated with modern cancer vaccines or oncolytic viruses, symptom-triggered evaluation remains appropriate, as the majority of reported cardiac complications occur in the context of systemic inflammatory responses or combination immunotherapy. In individuals with preexisting cardiovascular disease, prior exposure to cardiotoxic agents, or those receiving vaccine/OV + ICI combinations, a baseline ECG may be considered to establish a reference for subsequent comparison (Moey et al., 2021).

When symptoms such as chest pain, dyspnea, palpitations, syncope, unexplained fatigue, or hypotension arise, clinicians should promptly initiate a targeted cardiac workup. Initial evaluation typically includes ECG, high-sensitivity troponin, and natriuretic peptides to screen for myocarditis, arrhythmias, or early ventricular dysfunction. Transthoracic echocardiography should be performed when biomarker elevations, abnormal ECG findings, or hemodynamic instability are present, allowing assessment of ventricular function, wall motion, and pericardial effusion. In cases where myocarditis remains suspected, but echocardiography is nondiagnostic, cardiac MRI using updated Lake Louise criteria can provide further characterization and help distinguish inflammatory injury from ischemic or stress-induced cardiomyopathy. Diagnostic pathways should mirror those used for immune-related myocarditis caused by ICIs, as clinical and imaging phenotypes can overlap significantly (Murtagh et al., 2024).

#### 3.4.4 Management strategies

Management of cardiac toxicity from cancer vaccines and OVs is guided by the severity and underlying mechanism of injury. When immune-mediated myocarditis is suspected or confirmed, the foundational approach includes immediate discontinuation of the offending vaccine or viral therapy and initiation of high-dose corticosteroids, typically intravenous methylprednisolone, followed by a taper once clinical improvement is achieved (Lobenwein et al., 2021). In steroid-refractory cases, additional immunosuppressive therapies such as mycophenolate mofetil, IVIG, or anti-thymocyte globulin may be considered, borrowing from established protocols for ICI-induced myocarditis (Heem et al., 2024).

#### 3.4.5 Gaps in knowledge and future directions

Despite generally favorable cardiac safety profiles, several important knowledge gaps remain regarding vaccine- and OV-associated cardiotoxicity. A major challenge is the lack of prospective cardiac surveillance in clinical trials, limiting precise estimates of incidence and hindering the identification of subclinical or delayed cardiac effects. Similarly, mechanistic understanding remains incomplete: distinguishing cytokine-mediated myocardial depression, granulomatous hypersensitivity, and true viral or immune-mediated myocarditis is often challenging, particularly in combination regimens where overlapping toxicities may confound attribution.

Long-term cardiovascular outcomes after OV or vaccine exposure especially in the era of multimodal immunotherapy are also poorly characterized. It is unknown whether these therapies may contribute to cumulative inflammatory injury or interact adversely with preexisting atherosclerosis or subclinical cardiomyopathy. Moreover, standardized guidelines for monitoring, risk stratification, and management across diverse vaccine and viral platforms are lacking. Future research should prioritize prospective registries, harmonized cardiac endpoints, and translational studies examining immune signatures associated with cardiotoxicity. As vaccines, viral immunotherapies, and ICI combinations continue to evolve, deeper mechanistic and clinical insights will be essential for optimizing safety while preserving antitumor efficacy.

### 3.5 Emerging immunotherapy targets: TIGIT and CD47/SIRPα blockade

Several next-generation immune targets including TIGIT and CD47/SIRPα remain investigational, and cardiovascular safety data are currently insufficient to justify detailed mechanistic or management frameworks comparable to approved ICIs (Zhang et al., 2025). For TIGIT blockade, the most clinically advanced program (Tiragolumab) experienced major phase III setbacks, including discontinuation of at least one late-stage study and failure to meet key survival endpoints, leaving TIGIT agents unapproved and limiting interpretability of rare cardiac events reported in early studies.

For CD47/SIRPα blockade, Magrolimab has undergone program modifications with ongoing safety monitoring and discontinuation of specific phase III efforts, underscoring the evolving risk–benefit landscape for this class. Until robust prospective datasets with adjudicated cardiovascular endpoints become available, clinicians should consider trial-based safety protocols and maintain a low threshold for cardiac evaluation when systemic inflammatory toxicity occurs.

#### 3.5.1 Reported cardiotoxicities, epidemiology and risk factors

TIGIT (T-cell immunoreceptor with Ig and ITIM domains) is an inhibitory receptor expressed on activated T cells and NK cells and has been pursued most commonly in combination with PD-1/PD-L1 inhibitors to enhance antitumor immunity. However, TIGIT-directed therapies remain investigational, and the current cardiovascular safety dataset is insufficient to support mechanistic or management frameworks comparable to approved ICIs (Zhang et al., 2025). Notably, the most clinically advanced TIGIT program (tiragolumab) has faced major phase III setbacks, including discontinuation of at least one late-stage study and failure to meet key survival endpoints, which limits interpretability of rare cardiac events reported in early-phase experience (Assets.Roche, 2024). In that context, myocarditis attributed to TIGIT blockade appears exceptionally uncommon and when described typically occurs alongside multisystem irAEs (e.g., concurrent myositis, hepatitis, or neurologic toxicity), suggesting a broader immune activation phenotype rather than an isolated cardiac syndrome (Yang et al., 2023).

CD47 is a “do not eat me” signal that inhibits macrophage-mediated phagocytosis through the SIRPα receptor. Therapeutic blockade, with agents such as magrolimab, enhances macrophage recognition and clearance of tumor cells. In addition, the magrolimab program has undergone modifications with ongoing safety monitoring and discontinuation of specific phase III efforts, underscoring the shifting risk–benefit landscape for this class. Unlike T-cell checkpoints, CD-47 inhibition primarily modulates the innate immune system, with downstream effects on macrophage activation, erythrophagocytosis, and vascular homeostasis. Clinical data have shown reductions in vascular inflammation on imaging, and so far, no consistent signal for myocarditis or serious cardiac immune toxicity has emerged. Nevertheless, careful surveillance remains necessary, as macrophages play essential roles in cardiac remodeling, inflammation, and conduction-system biology (Li et al., 2023).

Epidemiologic data on cardiotoxicity from emerging immunotherapies such as LAG-3, TIGIT, and CD-47/SIRPα inhibitors remain limited, largely because current evidence derives from early-phase trials with relatively small patient cohorts and minimal systematic cardiac surveillance. Nevertheless, several risk patterns are beginning to emerge. Cardiovascular adverse events appear more likely to occur in the context of combination regimens, particularly those pairing LAG-3 or TIGIT blockade with PD-1/PD-L1 inhibitors, reflecting the heightened autoimmune activation associated with dual checkpoint inhibition. Patients with preexisting cardiovascular disease may be especially vulnerable to inflammatory or autoimmune myocardial injury, while cases of myocarditis that have been reported often coincide with multisystem immune-related adverse events including myositis, hepatitis, or neurologic syndromes suggesting a broader systemic immune activation rather than isolated cardiac involvement. Additional factors such as older age, baseline autoimmune disorders, and prior exposure to cardiotoxic therapies including anthracyclines or thoracic radiation may further increase susceptibility, although formal risk prediction models have not yet been established. Importantly, accurately determining true incidence remains challenging due to the lack of systematic troponin monitoring, variability in reporting practices across studies, and the absence of uniformly adjudicated cardiac endpoints (Stefanou et al., 2025).

#### 3.5.2 Proposed mechanisms of cardiotoxicity

Because TIGIT blockade and CD-47/SIRPα blockade remain investigational and are not approved for routine clinical use, the current clinical evidence base is insufficient to support a detailed discussion of cardiotoxicity mechanisms or to justify agent-specific diagnosis, monitoring, or management frameworks. Any proposed cardiovascular biology for these agents is therefore largely hypothesis-generating and extrapolated from broader immuno-oncology principles (e.g., intensified immune activation with combinatorial checkpoint blockade or macrophage-targeted modulation) rather than derived from robust human datasets with adjudicated cardiac endpoints. Accordingly, this section should be interpreted as a cautionary note: reported cardiac events are difficult to contextualize given small sample sizes, heterogeneous reporting, and the absence of prospective cardiovascular surveillance in most studies, underscoring the need for more definitive, systematically collected human safety data before mechanistic or clinical guidance can be responsibly proposed. Although these consequences have not been clinically validated, mechanistic plausibility warrants continued evaluation (Sharma et al., 2024; Shan C. et al., 2025).

### 3.6 Sex differences and cardio-immunotoxicity: an emerging but under-resolved variable

Sex-based immune differences influence antitumor immunity and immune-related adverse event (irAE) patterns and may plausibly affect cardio-immunotoxicity risk (St Clair et al., 2023). Across immune checkpoint inhibitor studies, women often exhibit different irAE profiles than men, but cardiac irAEs are rare and frequently underpowered for sex-stratified inference (Shan W. et al., 2025). Available analyses suggest that sex can modify toxicity and treatment response, yet definitive conclusions for myocarditis, thrombosis, and heart failure phenotypes are limited by confounding (tumor type, regimen intensity, baseline cardiovascular risk, and reporting heterogeneity) (Wilcox et al., 2022). Prospective cardio-oncology registries and trials should routinely capture sex-stratified cardiovascular endpoints and integrate sex as a prespecified variable in risk prediction models.

## 4 Conclusion

The rapidly evolving immunotherapy landscape has transformed cancer outcomes, improved survival, and enabled durable remission across multiple malignancies. As immune checkpoint inhibitors, CAR-T cell therapies, and bispecific T-cell engagers move into earlier lines of treatment and are increasingly used in combination, their impact on the heart cannot be treated as an afterthought. Cardiovascular toxicity is no longer a rare surprise, it is a predictable risk that we need to anticipate and manage if we want patients to benefit fully and safely from these powerful therapies.

Early identification and management of cardiotoxicity require a deliberate, structured approach that integrates clinical risk stratification with biomarkers, advanced imaging, and standardized surveillance pathways. Baseline evaluation, targeted monitoring during high-risk treatment windows, and timely diagnostic work-up for new symptoms or biomarker changes are essential to detect injury at a subclinical stage and intervene before irreversible damage occurs. Integrating cardio-oncology expertise into multidisciplinary tumor boards and treatment planning allows clinicians to balance cancer control with individualized cardiovascular risk, rather than viewing cardiotoxicity as an unavoidable downstream consequence.

Looking ahead, further progress in studies can clarify how immune therapies injure the heart, while real-world registries and clinical trials with standardized cardiovascular endpoints will help define who is most at risk and what strategies work best. At the same time, there is a need to refine risk prediction tools, incorporate digital health and remote monitoring, and test cardioprotective approaches that do not undermine the anti-tumor effects of immunotherapy. Providing practical training for oncologists, cardiologists, and trainees can help ensure that emerging evidence is translated into everyday clinical care.

Ultimately, the goal is not to limit the use of transformative cancer immunotherapies, but to deliver them more safely and equitably. Through sustained interdisciplinary collaboration among oncologists, cardiologists, immunologists, imaging specialists, and basic scientists, we can preserve the remarkable therapeutic benefits of immunotherapy while safeguarding cardiovascular health. In doing so, cardio-oncology will play a crucial role in ensuring that the promise of immunotherapy translates into longer, healthier survivorship for patients.
